# Voxel-Based Analysis of the Relation of 3′-Deoxy-3′-[^18^F]fluorothymidine ([^18^F]FLT) PET and Diffusion-Weighted (DW) MR Signals in Subcutaneous Tumor Xenografts Does Not Reveal a Direct Spatial Relation of These Two Parameters

**DOI:** 10.1007/s11307-021-01673-2

**Published:** 2021-11-09

**Authors:** Sonja Schelhaas, Lynn Johann Frohwein, Lydia Wachsmuth, Sven Hermann, Cornelius Faber, Klaus P. Schäfers, Andreas H. Jacobs

**Affiliations:** 1 European Institute for Molecular Imaging (EIMI), Westfälische Wilhelms-Universität Münster, Waldeyerstr. 15, 48149 Münster, Germany; 2 Translational Research Imaging Center, Clinic of Radiology, University Hospital of Münster, Münster, Germany; 3 Department of Geriatric Medicine, Johanniter Hospital, Bonn, Germany

**Keywords:** [^18^F]FLT PET, DW-MRI, Preclinical imaging, Oncology imaging, Voxel-analysis

## Abstract

**Purpose:**

Multimodal molecular imaging allows a direct coregistration of different images, facilitating analysis of the spatial relation of various imaging parameters. Here, we further explored the relation of proliferation, as measured by [^18^F]FLT PET, and water diffusion, as an indicator of cellular density and cell death, as measured by diffusion-weighted (DW) MRI, in preclinical tumor models. We expected these parameters to be negatively related, as highly proliferative tissue should have a higher density of cells, hampering free water diffusion.

**Procedures:**

Nude mice subcutaneously inoculated with either lung cancer cells (*n* = 11 A549 tumors, *n* = 20 H1975 tumors) or colorectal cancer cells (*n* = 13 Colo205 tumors) were imaged with [^18^F]FLT PET and DW-MRI using a multimodal bed, which was transferred from one instrument to the other within the same imaging session. Fiducial markers allowed coregistration of the images. An automatic post-processing was developed in MATLAB handling the spatial registration of DW-MRI (measured as apparent diffusion coefficient, ADC) and [^18^F]FLT image data and subsequent voxel-wise analysis of regions of interest (ROIs) in the tumor.

**Results:**

Analyses were conducted on a total of 76 datasets, comprising a median of 2890 data points (ranging from 81 to 13,597). Scatterplots showing [^18^F]FLT vs. ADC values displayed various grades of relations (Pearson correlation coefficient (PCC) varied from − 0.58 to 0.49, median: -0.07). When relating PCC to tumor volume (median: 46 mm^3^, range: 3 mm^3^ to 584 mm^3^), lung tumors tended to have a more pronounced negative spatial relation of [^18^F]FLT and ADC with increasing tumor size. However, due to the low number of large tumors (> ~ 200 mm^3^), this conclusion has to be treated with caution.

**Conclusions:**

A spatial relation of water diffusion, as measured by DW-MRI, and cellular proliferation, as measured by [^18^F]FLT PET, cannot be detected in the experimental datasets investigated in this study.

**Supplementary Information:**

The online version contains supplementary material available at 10.1007/s11307-021-01673-2.

## Introduction

In the recent years, molecular imaging has vastly contributed to the understanding of tumor biology. Besides providing quantitative readouts of physiological parameters, it can provide these in a spatially resolved manner, and imaging can be performed repeatedly, facilitating visualization of disease progression or response to therapy. Comparison of molecular imaging results with immunohistochemical staining of respective tissue samples showed that imaging parameters indeed reflect the tumor biology [1]. Imaging is often not only conducted with a single modality. For instance, positron emission tomography (PET) is frequently combined with computed tomography (CT), complementing the molecular PET results with morphological CT data. This not only allows the exact anatomical definition of regions of interest (ROIs) but can also contribute to improved image correction by facilitating attenuation correction [2]. The use of combined PET and magnetic resonance imaging (MRI) is becoming more frequent, as the number of hybrid PET and MR instruments is increasing. MR does not only provide information on anatomy, with good soft tissue contrast, but it can also provide microstructural and cellular information. Diffusion-weighted MRI measures the apparent diffusion coefficient (ADC) as an indicator for tissue integrity and can indicate cell death. As induction of cell death is a common therapeutic approach, this imaging parameter can be used to follow therapy response [3]. Therefore, ADC mapping has been suggested to be suitable as response assessment marker in the treatment of gliomas [4, 5].

Another molecular imaging approach often used to monitor tumor therapy is PET using the radiotracer 3′-deoxy-3′-[^18^F]fluorothymidine ([^18^F]FLT). [^18^F]FLT is a thymidine analog; hence, its uptake reflects cellular proliferation, and it has been successfully used to monitor therapy response both preclinically [6] and clinically [7]. As cellular proliferation is often decreased in areas of cell death, one could assume a negative spatial correlation of [^18^F]FLT and ADC. However, a potential relation of these two imaging parameters has only scarcely been explored.

Here, we reanalyzed previously published data [8, 9] of subcutaneous tumor xenografts imaged with [^18^F]FLT PET and DW-MRI to explore the relation of these two readouts using a voxel-based approach.

## Materials and Methods

### Tumor Models and Image Acquisition

Images from previously published tumor-bearing mice were reanalyzed. Here, we focus on the vehicle-treated tumors, or on tumors before treatment initiation, to assess [^18^F]FLT and ADC relation in untreated tumors.

In brief, 2 × 10^6^ lung cancer cells (A549 and H1975 [8]) were inoculated in NMRI nude mice. Vehicle treatment (for comparison to gemcitabine treatment) was conducted by intraperitoneal injection of 2.5 µl/g bodyweight 0.9% NaCl and PET, and MR imaging was performed on the following day (d1), as well as before treatment initiation (d-6). For the colorectal cancers [9], 5 × 10^6^ Colo205 were implanted in CD1 nude mice, and vehicle treatment (for comparison to a FOLFOX-like therapy; 0 h: 0.9% NaCl 1.6 µl/g bodyweight i.p.; 1.5 h: 5% glucose 9 µl/g bodyweight retrobulbar; 2 h and 6 h: 0.9% NaCl 0.6 µl/g bodyweight) was done on d0 and d7. PET and MR imaging were performed baseline (d-1) and on d1, d2, d6, d9, and d13. The electronic supplementary data reveals details on the imaging schedules of individual tumors.

PET images were acquired with a quadHIDAC camera (Oxford Positron Systems, Oxfordshire) 70–90 min after injection of ~ 10 MBq [^18^F]FLT. During the tracer uptake period, T2-weighted (T2w) MR and DW-MR images were obtained using a 9.4 T small animal MRI scanner (Bruker Biospin GmbH, Ettlingen, Germany). T2w images were acquired with 2D rapid acquisition with relaxation enhancement (RARE), TR/TE 3600/40 ms, RARE factor 8, FOV 35 mm, 256 matrix, followed by DW images (EPI-DTI), TR/TE 1000/19 ms, 12 segments, 7 b-values from 0 to 700 s/mm^2^, 128 matrix, NEX 6, respiration-triggered. Four slices at biggest tumor diameters were measured with DW-MR with a slice thickness of 1 mm each. An in-house developed PET-MRI animal bed was utilized to allow coregistration of the two modalities by use of fiducial markers filled with water-diluted PET tracer, being visible in both PET and MRI.

### Image Analysis

[^18^F]FLT was calculated as %ID/ml using the in-house developed software MEDgical. ADC was calculated using a monoexponential fit as described in detail previously [8, 9]. In order to assess the correlation between [^18^F]FLT-PET and ADC-MRI data, a voxel-wise analysis in MATLAB (The MathWorks, Natick, MA, USA) has been developed.

In a first step, [^18^F]FLT data were manually coregistered to the MRI datasets using the fiducial markers of the common animal bed. Then, a region of interest (ROI) was manually defined on the anatomical MRI dataset. As ADC data were acquired with a small field of view and thus with very few voxels, they were interpolated using nearest-neighbor interpolation to preserve the measured ADC values. The ROI was then used to extract the voxel intensities from [^18^F]FLT and ADC datasets in a voxel-wise manner. To assess the validity of our analyses, we applied variations to the coregistration and analyzed the respective relation of [^18^F]FLT and ADC in a few datasets (by the same reader). We could not detect any substantial changes in our analyses. Also variations in the manual definitions of the ROIs did not affect the data considerably.

### Statistical Analysis

The Pearson correlation coefficients (PCCs) were calculated using the software SigmaPlot (version 13.0). *P*-values < 0.05 were considered statistically significant.

## Results

**Fig. 1:**
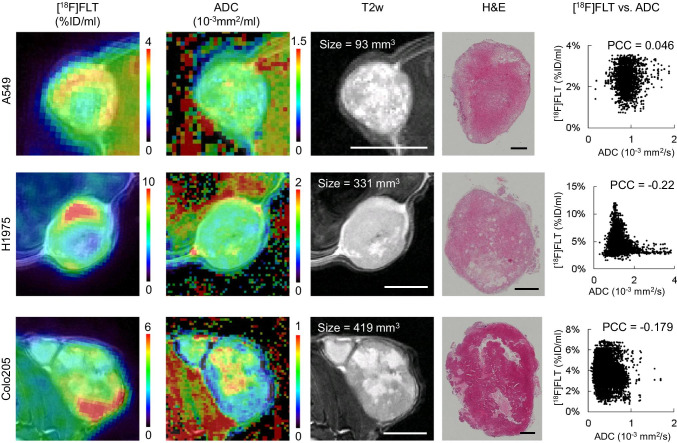
Exemplary images of coregistered [^18^F]FLT PET and DW-MRI show varying degrees of relation of these two parameters. [^18^F]FLT PET images (first column), DW-MR images (second column), as well as T2w MR images (third column) of a transverse tumor section are shown for A549 (first row), H1975 (second row), and Colo205 (third row). Hematoxylin and eosin staining (H&E) images are depicted for morphological reference. The last column shows scatterplots of the calculated relation of [^18^F]FLT and ADC of the whole tumor analyzed. Scale bar on T2w images = 5 mm. Scale bar on H&E images = 1 mm

**Fig. 2:**
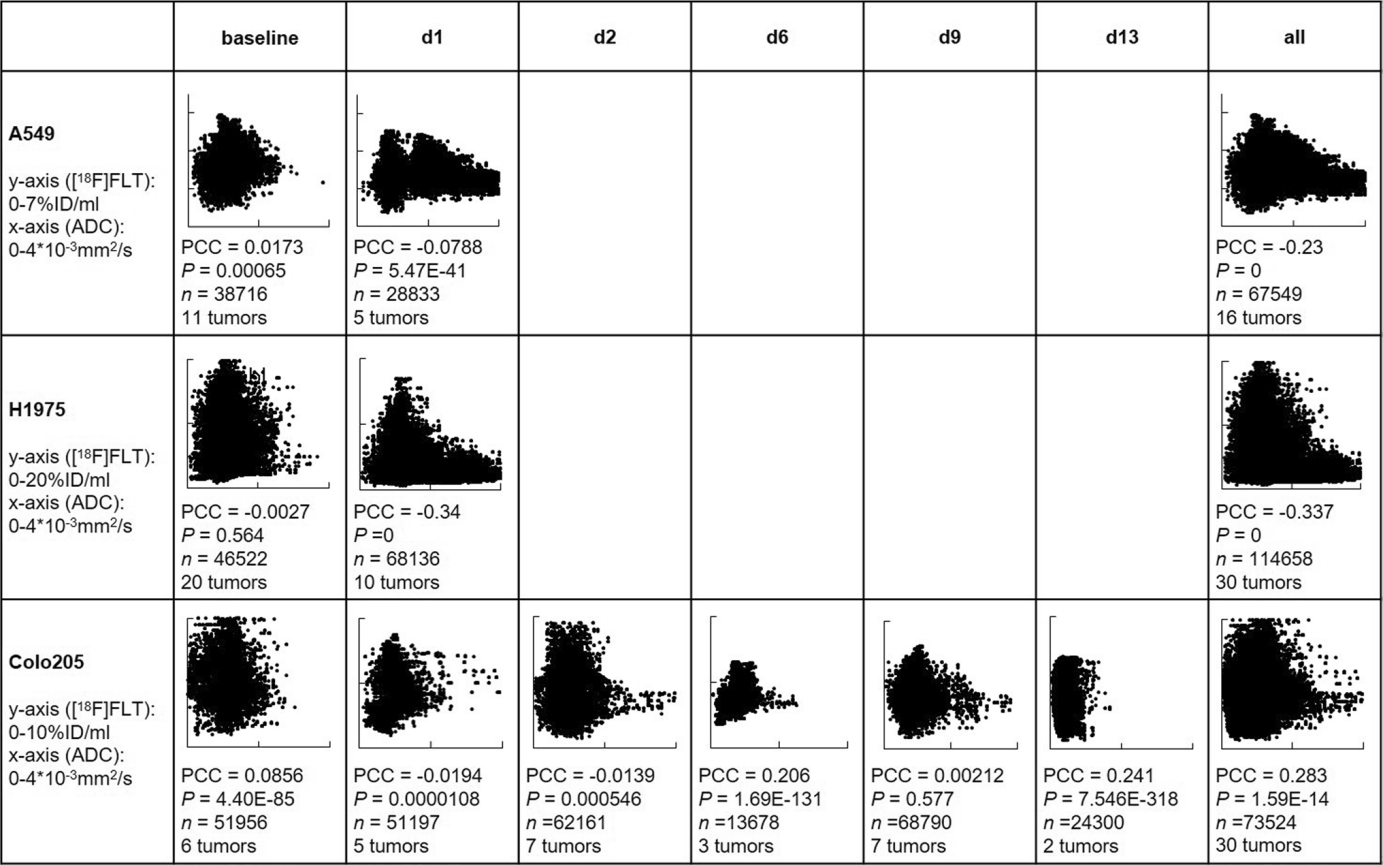
Summarized results of the voxel-analysis performed here

**Fig. 3:**
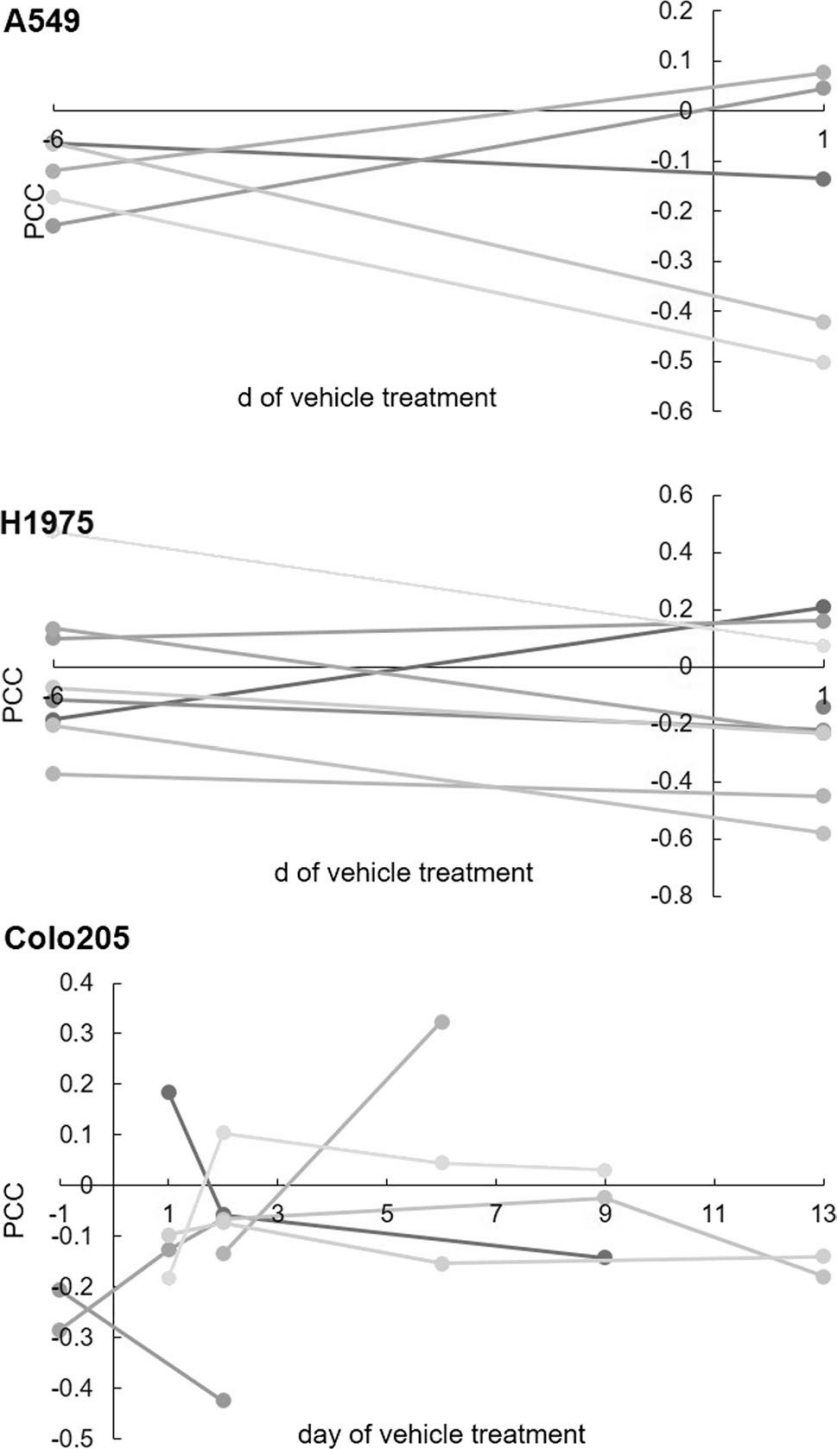
PCC is not directly related to tumor growth. PCCs of the tumors that were imaged repeatedly during vehicle treatment were related to the day of treatment to investigate how the spatial relation of [^18^F]FLT and ADC is affected while the individual tumors grow. Every single gray line represents an individual tumor analyzed (*n* = 5 for A549, *n* = 8 for H1975, *n* = 6 for Colo205)

**Fig. 4:**
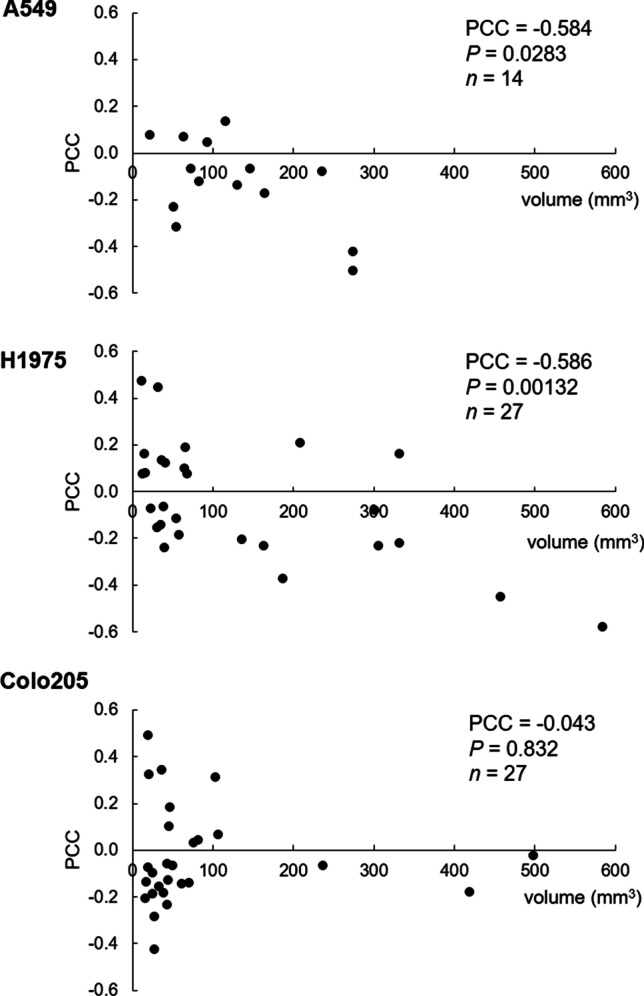
PCCs of [^18^F]FLT vs. ADC plots of all tumors analyzed were plotted against the tumor volume as determined by MRI

A total of 76 datasets were analyzed, comprising a median of 2890 data points (ranging from 81 to 13,597), from a total of 44 tumors, ranging in size from 3 to 584 mm^3^ (median: 46 mm^3^). [^18^F]FLT vs. ADC data were plotted and revealed various degrees of relation. The Pearson correlation coefficient was calculated as an indicator of correlation, in analogy to previously published data [10, 11]. Figure 1 shows one example per tumor cell line, comprising not only the respective scatterplots, but also a transverse slice of [^18^F]FLT, ADC, T2w MRI, and hematoxylin and eosin staining (H&E) images. All scatterplots can be found in the supplementary tables. A summary of the individual analyses, sorted according to tumor type and day of imaging, is displayed in Fig. 2. PCC varied substantially (ranging from − 0.58 to 0.49, median: − 0.0657), and no clear relationship could be identified, irrespective of tumor type. As most of the tumors were measured repeatedly, we further analyzed the relation of the PCC relative to the day of imaging (Fig. 3). Again, no straightforward relationship was noted. As a next step, we tried to investigate a potential relation of PCC to tumor volume (Fig. 4). While there was no relation visible in the colorectal cancers, we noted a statistically significant correlation of PCC with tumor volume in the two lung cancer models A549 (PCC =  − 0.584, *P* = 0.028, *n* = 14 tumors) and H1975 (PCC =  − 0.586, *P* = 0.001, *n* = 27 tumors). In contrary, analysis of the relative changes (i.e., delta) did not reveal any relation (see Supplementary Fig. 1).

## Discussion

We here report a voxel-by-voxel analysis of [^18^F]FLT and ADC data obtained from various preclinical subcutaneous tumors. We expected to detect increased water diffusion (as measured by ADC) in areas of low proliferation (as measured by [^18^F]FLT). We could observe this relationship only in few lung cancer xenografts with a volume above ~ 200 mm^3^. Larger tumors tend to be more heterogeneous, which could contribute to a negative spatial relation. However, due to the low number of larger tumors, this relationship has to be treated with caution. The majority of the tumors analyzed showed varying degrees of relation with no apparent relation to tumor growth or tumor type.

Our results, originating from a range of preclinical tumors, are in line with previous reports that compared [^18^F]FLT and ADC data on a voxel basis in single-tumor entities. Honndorf et al. calculated the PCC between ADC and [^18^F]FLT or [^18^F]FDG in treated colorectal HCT116 xenografts and compared that to vehicle treated tumors [10]. During vehicle treatment, PCC decreased significantly, which was in line with increasing tumor volumes, which were evolving from ~ 300 to 900 mm^3^ on day 7. Unfortunately, a direct relation of PCC to individual tumor volumes was not performed, so a direct comparison to our data is not possible. However, in a previous study by the same group, using the same tumor model, no significant correlation of the [^18^F]FLT vs. ADC scatterplots could be observed in vehicle treated tumors, despite substantial tumor growth over 8 days (from ~ 300 to ~ 700 mm^3^) [11], implying that the relation of PCC to tumor growth or volume is not straightforward.

A clinical study in twelve non-small cell lung cancer patients also showed that [^18^F]FLT was not correlated with ADC and that the spatial distribution of aggressive areas did not reveal any systemic relation [12].

Some further clinical reports investigated the spatial relation of glucose metabolism ([^18^F]FDG) and water diffusivity (ADC). In breast cancer patients, the negative correlation of these two imaging parameters was statistically significant (PCC =  − 0.39 ± 0.24 in untreated tumors, *n* = 7 [13]). And also in lung cancer patients, a significant negative correlation of [^18^F]FDG and ADC was observed (*n* = 5, ranging from − 0.41 to − 0.17) [14]. On the other hand, another study showed that DW-MRI and [^18^F]FDG PET in metastatic non-small cell lung cancer were not necessarily correlated in all tumor areas [15].

[^18^F]FDG PET and MR ADC histogram metrics in pediatric diffuse intrinsic pontine glioma demonstrate different characteristics with often a negative correlation between PET and MR ADC pixel values. A higher negative correlation was associated with lower progression-free survival, which may indicate higher-grade elements within the tumor [16].

Hence, our data confirm that the spatial relation of cellular proliferation as measured by [^18^F]FLT PET and cell death, as measured by DW-MRI, are not straightforward. This might in part be due to the limitations of the spatial resolution of these two imaging approaches as well as to heterogeneous tumor growth and tissue compartments in tumors. On the other hand, proliferation induced by cell death [17, 18] might hamper a strict negative spatial relation of these two parameters.

## Supplementary Information

Below is the link to the electronic supplementary material.

Supplementary file1 (PDF 173 KB)

## References

[1] García-Figueiras R, Baleato-González S, Padhani AR (2019). How clinical imaging can assess cancer biology. Insights Imaging.

[2] Brady SL, Shulkin BL (2017). Dose optimization: a review of CT imaging for PET attenuation correction. Clin Transl Imaging.

[3] Sinkus R, Van Beers BE, Vilgrain V (2012). Apparent diffusion coefficient from magnetic resonance imaging as a biomarker in oncology drug development. Eur J Cancer.

[4] Hamstra DA, Chenevert TL, Moffat BA (2005). Evaluation of the functional diffusion map as an early biomarker of time-to-progression and overall survival in high-grade glioma. Proc Natl Acad Sci U S A.

[5] Moffat BA, Chenevert TL, Lawrence TS (2005). Functional diffusion map: a noninvasive MRI biomarker for early stratification of clinical brain tumor response. Proc Natl Acad Sci U S A.

[6] S Schelhaas K Heinzmann VR Bollineni et al 2017 Preclinical applications of 3’-deoxy-3’-[18F]fluorothymidine in oncology - a systematic review Theranostics 710.7150/thno.16676 10.7150/thno.16676 PMC5196884 28042315

[7] Bollineni VR, Kramer GM, Jansma EP (2016). A systematic review on [18F]FLT-PET uptake as a measure of treatment response in cancer patients. Eur J Cancer.

[8] S Schelhaas A Held L Wachsmuth et al 2016 Gemcitabine mechanism of action confounds early assessment of treatment response by 3′-deoxy-3′-[18F]fluorothymidine in preclinical models of lung cancer Cancer Res 7610.1158/0008-5472.CAN-16-1479 10.1158/0008-5472.CAN-16-1479 27784748

[9] S Schelhaas L Wachsmuth S Hermann et al 2018 Thymidine metabolism as a confounding factor for 3’-deoxy-3’-18F-fluorothymidine uptake after therapy in a colorectal cancer model J Nucl Med 5910.2967/jnumed.117.206250 10.2967/jnumed.117.206250 29476002

[10] Honndorf VS, Schmidt H, Wiehr S (2016). The synergistic effect of selumetinib/docetaxel combination therapy monitored by [(18) F]FDG/[(18) F]FLT PET and diffusion-weighted magnetic resonance imaging in a colorectal tumor xenograft model. Mol imaging Biol.

[11] Honndorf VS, Schmidt H, Wehrl HF, et al (2014) Quantitative correlation at the molecular level of tumor response to docetaxel by multimodal diffusion-weighted magnetic resonance imaging and [^18^F]FDG/[^18^F]FLT positron emission tomography. Mol Imaging 13 10.2310/7290.2014.00045 10.2310/7290.2014.00045 25430886

[12] Christensen TN, Langer SW, Villumsen KE (2020). 18F-fluorothymidine (FLT)-PET and diffusion-weighted MRI for early response evaluation in patients with small cell lung cancer: a pilot study. Eur J Hybrid Imaging.

[13] Ostenson J, Pujara AC, Mikheev A (2017). Voxelwise analysis of simultaneously acquired and spatially correlated 18F-fluorodeoxyglucose (FDG)-PET and intravoxel incoherent motion metrics in breast cancer. Magn Reson Med.

[14] Schmidt H, Brendle C, Schraml C (2013). Correlation of simultaneously acquired diffusion-weighted imaging and 2-deoxy-[18F] fluoro-2-D-glucose positron emission tomography of pulmonary lesions in a dedicated whole-body magnetic resonance/positron emission tomography system. Invest Radiol.

[15] S Metz C Ganter S Lorenzen et al 2015 Multiparametric MR and PET imaging of intratumoral biological heterogeneity in patients with metastatic lung cancer using voxel-by-voxel analysis PLoS One 1010.1371/journal.pone.0132386 10.1371/journal.pone.0132386 PMC4506136 26186719

[16] Zukotynski KA, Vajapeyam S, Fahey FH (2017). Correlation of ^18^ F-FDG PET and MRI apparent diffusion coefficient histogram metrics with survival in diffuse intrinsic pontine glioma: a report from the Pediatric Brain Tumor Consortium. J Nucl Med.

[17] Zimmerman MA, Huang Q, Li F (2013). Cell death-stimulated cell proliferation: a tissue regeneration mechanism usurped by tumors during radiotherapy. Semin Radiat Oncol.

[18] Fogarty CE, Bergmann A (2017). Killers creating new life: caspases drive apoptosis-induced proliferation in tissue repair and disease. Cell Death Differ.

