# Supplementary material for: Voxel-Based Analysis of the Relation of 3′-Deoxy-3′-[18F]fluorothymidine ([18F]FLT) PET and Diffusion-Weighted (DW) MR Signals in Subcutaneous Tumor Xenografts Does Not Reveal a Direct Spatial Relation of These Two Parameters
Source: Mol Imaging Biol. 2021 Nov 9;24(3):359–64. doi: 10.1007/s11307-021-01673-2 (PMC9085704; doi:10.1007/s11307-021-01673-2)
Supplement: Supplementary file 1 — Supplementary file1 (PDF 173 KB) [file 11307_2021_1673_MOESM1_ESM.pdf]

Electronic Supplementary Material:

Article: Voxel-based analysis of the relation of 3'-deoxy-3'-[^{18}F]fluorothymidine ([^{18}F]FLT) PET and diffusion weighted (DW) MR signals in subcutaneous tumor xenografts does not reveal a direct spatial relation of these two parameters

Journal: Molecular Imaging and Biology

Authors: [1] Sonja Schelhaas, [1] Lynn Johann Frohwein, [2] Lydia Wachsmuth, [1] Sven Hermann, [2] Cornelius Faber, [1] Klaus P. Schäfers, [1,3] Andreas H. Jacobs

[1] European Institute for Molecular Imaging (EIMI), Westfälische Wilhelms-Universität Münster, Münster, Germany.

[2] Translational Research Imaging Center, Clinic of Radiology, University Hospital of Münster, Münster, Germany

[3] Department of Geriatric Medicine, Johanniter Hospital, Bonn, Germany.

Corresponding author:

Dr. rer. nat. Sonja Schelhaas, European Institute for Molecular Imaging (EIMI), Waldeyerstr. 15, D-48149 Münster, Phone: +492518349312, Fax: +492518349313, email: sonja.schelhaas@uni-muenster.de

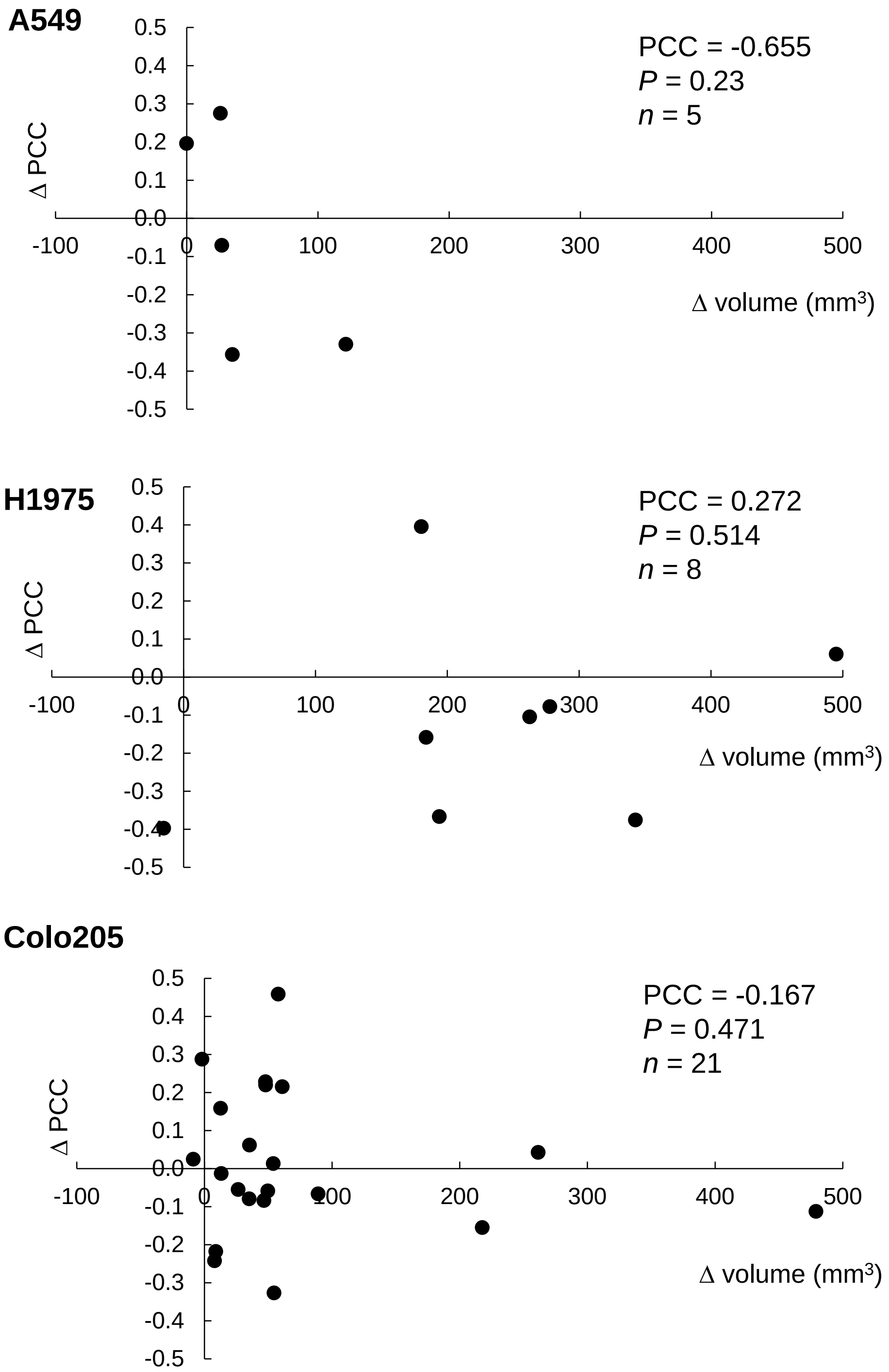

Supplementary Figure 1. Changes (Δ) of PCCs of [^{18}F]FLT vs. ADC plots of all tumors analyzed were plotted against respective changes in tumor volume.

Voxel analyses of the individual tumors of experimental A549 series 1
y-axis ([^{18}F]FLT): 0-8%ID/ml
x-axis (ADC): 0-4*10^{-3}mm^2/s

	baseline		d1	
6112L	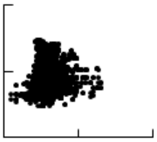	PCC = -0.064<br>*P* = 4.82E-04<br>*n* = 2972<br>72 mm^2	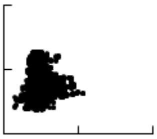	PCC = -0.135<br>*P* =9.01E-17<br>*n* = 3765<br>131 mm^2
6112R	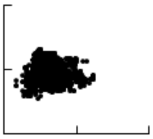	PCC = -0.229<br>*P* = 9.74E-25<br>*n* = 1955<br>51 mm^2	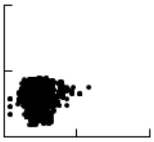	PCC = 0.046<br>*P* = 2.87E-03<br>*n* = 4191<br>93 mm^2
6113L	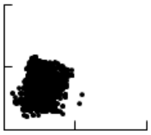	PCC = -0.119<br>*P* = 5.67E-14<br>*n* = 3942<br>82 mm^2	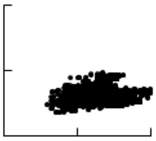	PCC = 0.077<br>*P* = 8.73E-08<br>*n* = 4823<br>21 mm^2
6113M	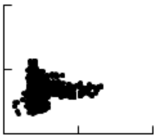	PCC = -0.0643<br>*P* = 4.25E-04<br>*n* = 3002<br>146 mm^2	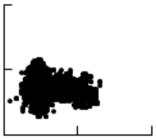	PCC = -0.421<br>*P* = 0.00E+00<br>*n* = 8679<br>274 mm^2
6113R	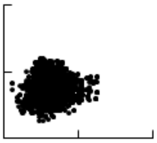	PCC = -0.172<br>*P* = 3.33E-37<br>*n* = 5420<br>164m m^2	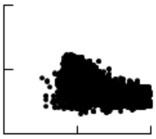	PCC = -0.502<br>*P* = 0.00E+00<br>*n* = 7375<br>274mm^2
6110L	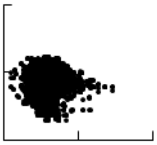	PCC = -0.0767<br>*P* = 4.05E-12<br>*n* = 8150<br>236 mm^2		
6110M	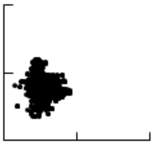	PCC = -0.314<br>*P* = 2.33E-51<br>*n* = 2194<br>55 mm^2		
6110R	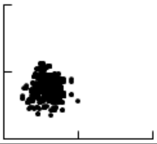	PCC = -0.0161<br>*P* = 0.653<br>*n* = 784<br>17 mm^2		
6111L	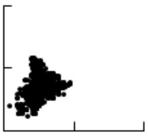	PCC = 0.0719<br>*P* = 1.83E-03<br>*n* = 1876<br>63 mm^2		
6111M	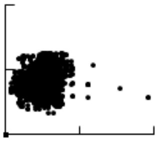	PCC = 0.136<br>*P* = 7.36E-20<br>*n* = 4481<br>115 mm^2		
6111R	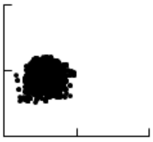	PCC = -0.0271<br>*P* = 0.0892<br>*n* = 3940<br>94 mm^2		

Voxel analyses of the individual tumors of experimental H1975 series 1

y-axis ([^{18}F]FLT): 0-20%ID/ml

x-axis (ADC): 0-4*$10^{-3}$$mm^2$/s

	baseline		d1	
5837L	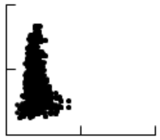	PCC =<br>-0.184<br>*P* =1.08E-20<br>*n* =2515<br>58 mm^2	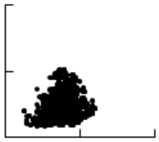	PCC =0.211<br>*P* =2.77E-59<br>*n* =5764<br>208 mm^2
5837R	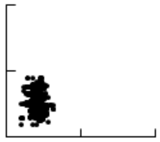	PCC =-0.0667<br>*P* =0.165<br>*n* =436<br>11 mm^2	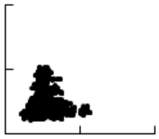	PCC =-0.141<br>*P* =5.75E-10<br>*n* =1922<br>36 mm^2
5838L	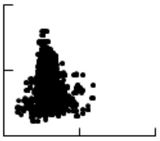	PCC =-0.115<br>*P* =5.69E-10<br>*n* =2890<br>54 mm^2	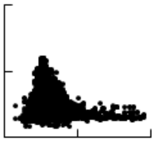	PCC =-0.22<br>*P* =1.81E-111<br>*n* =10115<br>331 mm^2
5838R	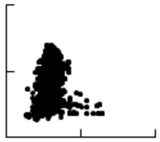	PCC =0.102<br>*P* =4.42E-08<br>*n* =2892<br>64 mm^2	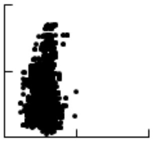	PCC =0.162<br>*P* =4.56E-49<br>*n* =8120<br>331 mm^2
5839L	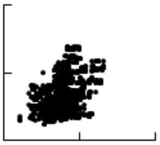	PCC =0.446<br>*P* =2.37E-197<br>*n* =4054<br>31 mm^2		
5839R	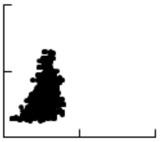	PCC =-0.154<br>*P* =2.75E-04<br>*n* =553<br>31 mm^2		
5840L	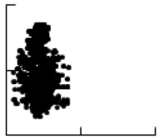	PCC =-0.0644<br>*P* =4.83E-03<br>*n* =1916<br>39 mm^2		
5840R	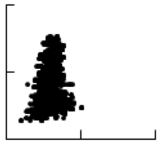	PCC = 0.122<br>*P* =5.69E-09<br>*n* =2268<br>41 mm^2		

Voxel analyses of the individual tumors of experimental H1975 series 2
y-axis ([^{18}F]FLT): 0-20%ID/ml
x-axis (ADC): 0-4*$10^{-3}mm^2/s$

	baseline		d 1	
5945L	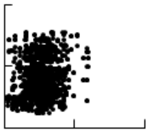	PCC = 0.136<br>*P* =5.70E-08<br>*n* =1588<br>36 mm^2	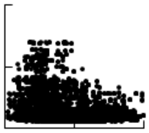	PCC = -0.231<br>*P* =1.49E-110<br>*n* =9078<br>305 mm^2
5945M	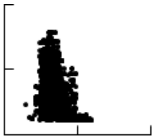	PCC = -0.372<br>*P* =3.15E-207<br>*n* = 6340<br>187 mm^2	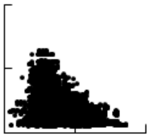	PCC = -0.45<br>*P* = 0.00E+00<br>*n* = 13070<br>458 mm^2
5945R	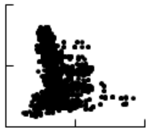	PCC = -0.204<br>*P* =4.56E-29<br>*n* =2959<br>136 mm^2	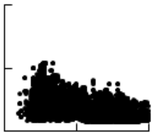	PCC = -0.58<br>*P* =0.00E+00<br>*n* =11290<br>584 mm^2
5951I	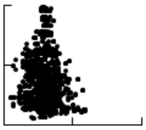	PCC = -0.0723<br>*P* =1.74E-03<br>*n* = 1872<br>23 mm^2	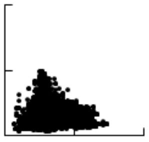	PCC = -0.231<br>*P* = 2.83E-69<br>*n* =5664<br>162 mm^2
5951M	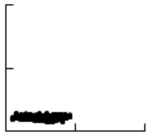	PCC = -0.0593<br>*P* =0.184<br>*n* = 504<br>8 mm^2	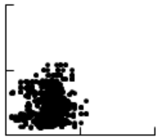	PCC =0.0588<br>*P* =0.0503<br>*n* = 1108<br>21 mm^2
5951R	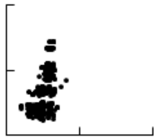	PCC = 0.474<br>*P* =1.85E-27<br>*n* = 466<br>12 mm^2	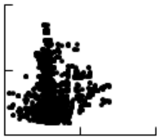	PCC = 0.0763<br>*P* = 6.28E-04<br>*n* = 2004<br>68 mm^2
5947L	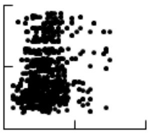	PCC = 0.188<br>*P* =1.78E-10<br>*n* =1135<br>65 mm^2		
5947M	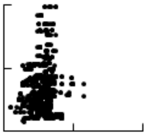	PCC = 0.0755<br>*P* = 2.07E-02<br>*n* = 938<br>12 mm^2		
5947R	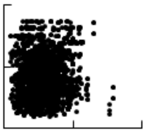	PCC = 0.0824<br>*P* = 2.33E-06<br>*n* = 3279<br>16 mm^2		
5949L	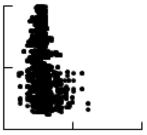	PCC = -0.238<br>*P* = 1.19E-28<br>*n* = 2120<br>40 mm^2		
5949M	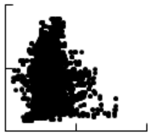	PCC = -0.0801<br>*P* = 6.28E-13<br>*n* = 8051<br>301 mm^2		
5949R	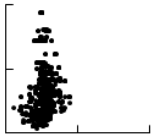	PCC = 0.163<br>*P* = 8.95E-07<br>*n* = 899<br>15 mm^2		

Voxel analyses of the individual tumors of experimental Colo205 series 1
y-axis ([^{18}F]FLT): 0-10%ID/ml
x-axis (ADC): 0-4*10^{-3}mm^2/s

	baseline	d1	d2	d9
8674R		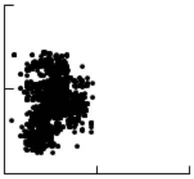<br>PPCC = 0.185<br>*P* = 3.84E-18<br>*n* =2158<br>46 mm^2	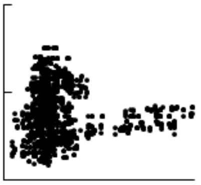<br>PCC = -0.0577<br>*P* = 1.29E-02<br>*n* =1857<br>43 mm^2	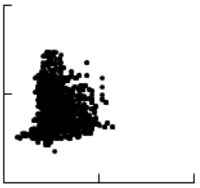<br>PCC =-0.142<br>*P* = 1.10E-13<br>*n* =2720<br>61 mm^2
8677L				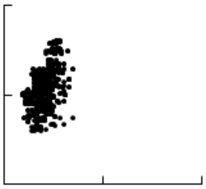<br>PCC = 0.494<br>*P* = 9.62E-101<br>*n* =1624<br>20 mm^2
8677R				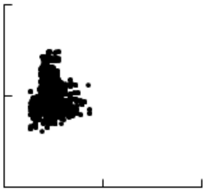<br>PCC = 0.315<br>*P* = 4.30E-89<br>*n* =3827<br>103 mm^2
8678L	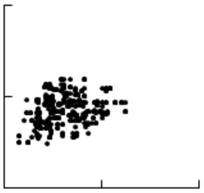<br>PCC = -0.206<br>*P* = 2.77E-13<br>*n* =1233<br>16 mm^2	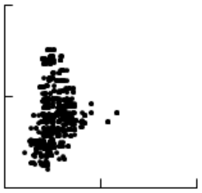<br>PCC = 0.0431<br>*P* = 9.19E-02<br>*n* =1531<br>15 mm^2	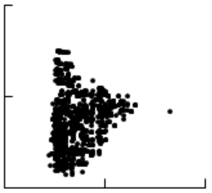<br>PCC = -0.424<br>*P* = 1.37E-91<br>*n* =2080<br>27 mm^2	
8678R	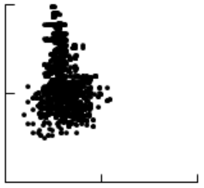<br>PCC = -0.285<br>*P* = 1.69E-45<br>*n* =2373<br>27 mm^2	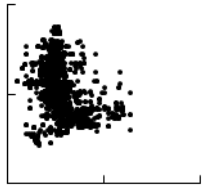<br>PCC = -0.127<br>*P* = 7.32E-11<br>*n* =2606<br>44 mm^2	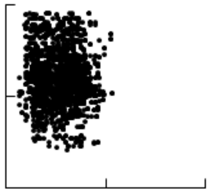<br>PCC = -0.0657<br>*P* = 4.06E-04<br>*n* =2891<br>50 mm^2	

Voxel analyses of the individual tumors of experimental Colo205 series 1

y-axis ($[^{18}F]FLT$): 0-10%ID/ml

x-axis (ADC): 0-4*$10^{-3}mm^2/s$

	baseline
8675L	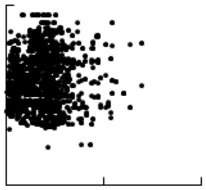<br>PCC = 0.0686<br>*P* = 3.94E-03<br>*n* =1766<br>107 mm^2
8675R	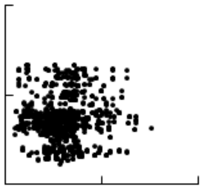<br>PCC = -0.185<br>*P* = 1.85E-13<br>*n* =1562<br>25 mm^2
8676L	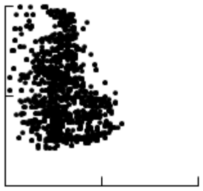<br>PCC = -0.233<br>*P* = 2.00E-30<br>*n* =2356<br>42 mm^2
8676R	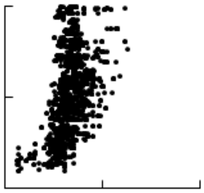<br>PCC = 0.346<br>*P* = 5.31E-50<br>*n* =1738<br>36 mm^2

Voxel analyses of the individual tumors of experimental Colo205 series 2
y-axis ($[^{18}F]$FLT): 0-10%ID/ml
x-axis (ADC): 0-4*10^{-3}mm^2/s

	d1	d2	d6	d9	d13
9144L		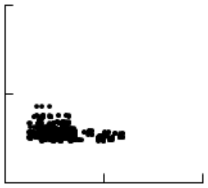<br>PCC = -0.134<br>*P* = 3.68E-11<br>*n* =2420<br>16 mm^2	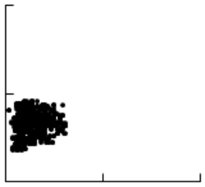<br>PCC = 0.324<br>*P* = 6.92E-67<br>*n* =2691<br>20 mm^2	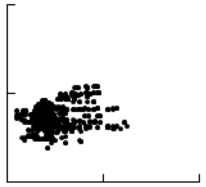<br>PCC = 0.023<br>*P* = 2.13E-01<br>*n* =2931<br>34 mm^2	
9144R		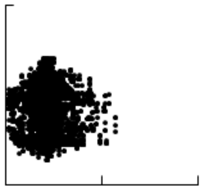<br>PCC = -0.0661<br>*P* = 5.94E-11<br>*n* =9784<br>236 mm^2		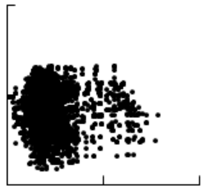<br>PCC = -0.0238<br>*P* = 1.15E-02<br>*n* =11250<br>498 mm^2	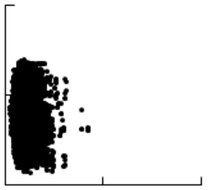<br>PCC = -0.179<br>*P* = 6.76E-98<br>*n* =13597<br>419 mm^2
9145L	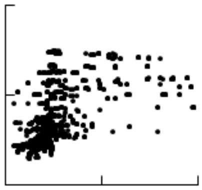<br>PCC = -0.0975<br>*P* = 3.70E-25<br>*n* =11250<br>25 mm^2	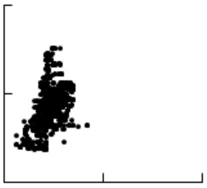<br>PCC = -0.0733<br>*P* = 1.43E-14<br>*n* =10985<br>19 mm^2	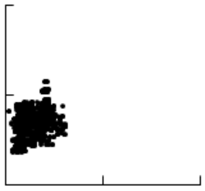<br>PCC = -0.153<br>*P* = 1.84E-59<br>*n* =11189<br>33 mm^2	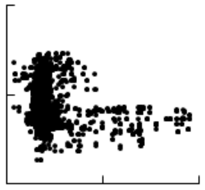<br>PCC = 0.011<br>*P* = 2.19E-01<br>*n* =12489<br>60 mm^2	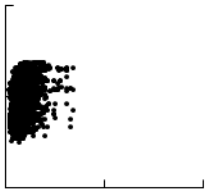<br>PCC = -0.14<br>*P* = 9.67E-56<br>*n* =12546<br>70 mm^2
9145R	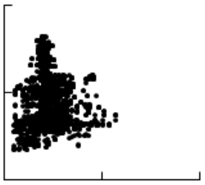<br>PCC = -0.183<br>*P* = 2.00E-07<br>*n* =11628<br>38 mm^2	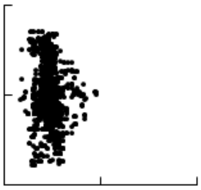<br>PCC = 0.104<br>*P* = 8.45E-30<br>*n* =11791<br>45 mm^2	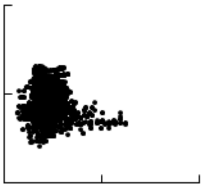<br>PCC = 0.0451<br>*P* = 2.44E-07<br>*n* =13115<br>81 mm^2	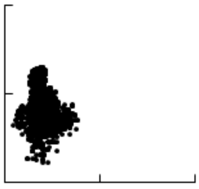<br>PCC = 0.0317<br>*P* = 2.17E-04<br>*n* =13597<br>76 mm^2	
